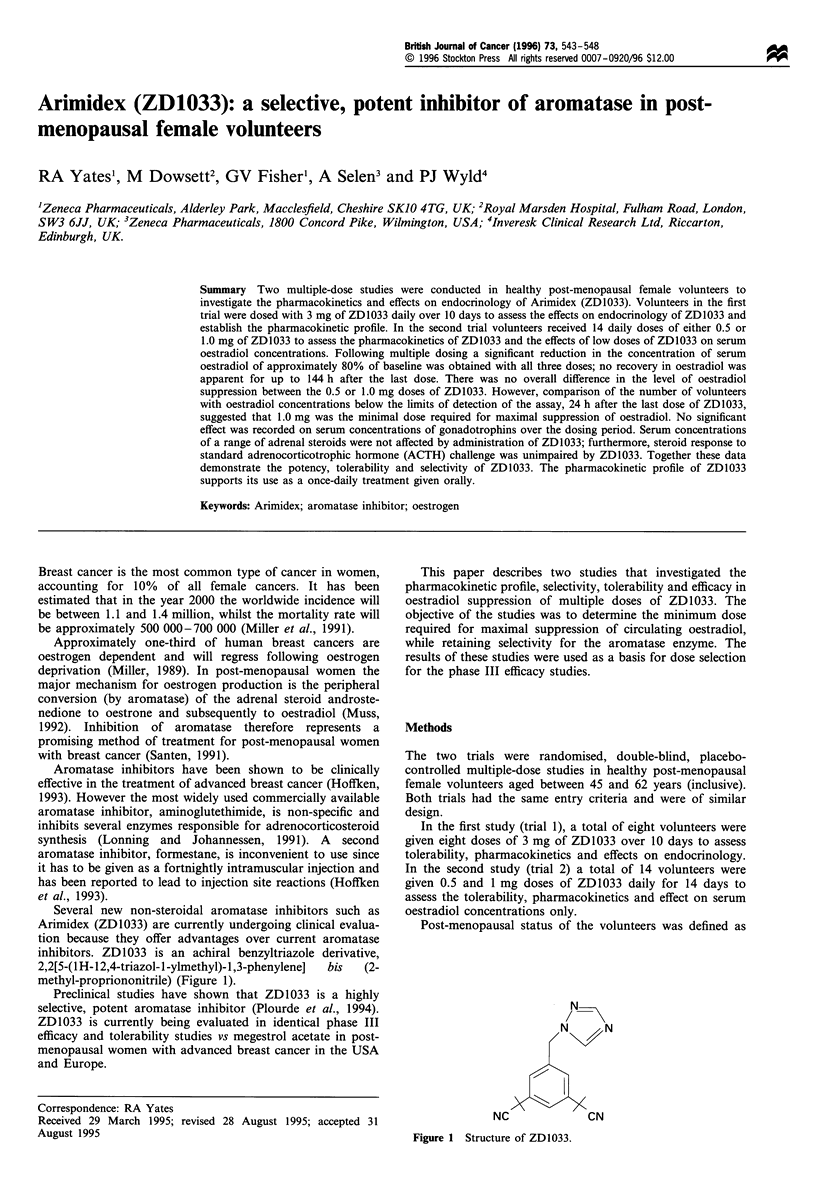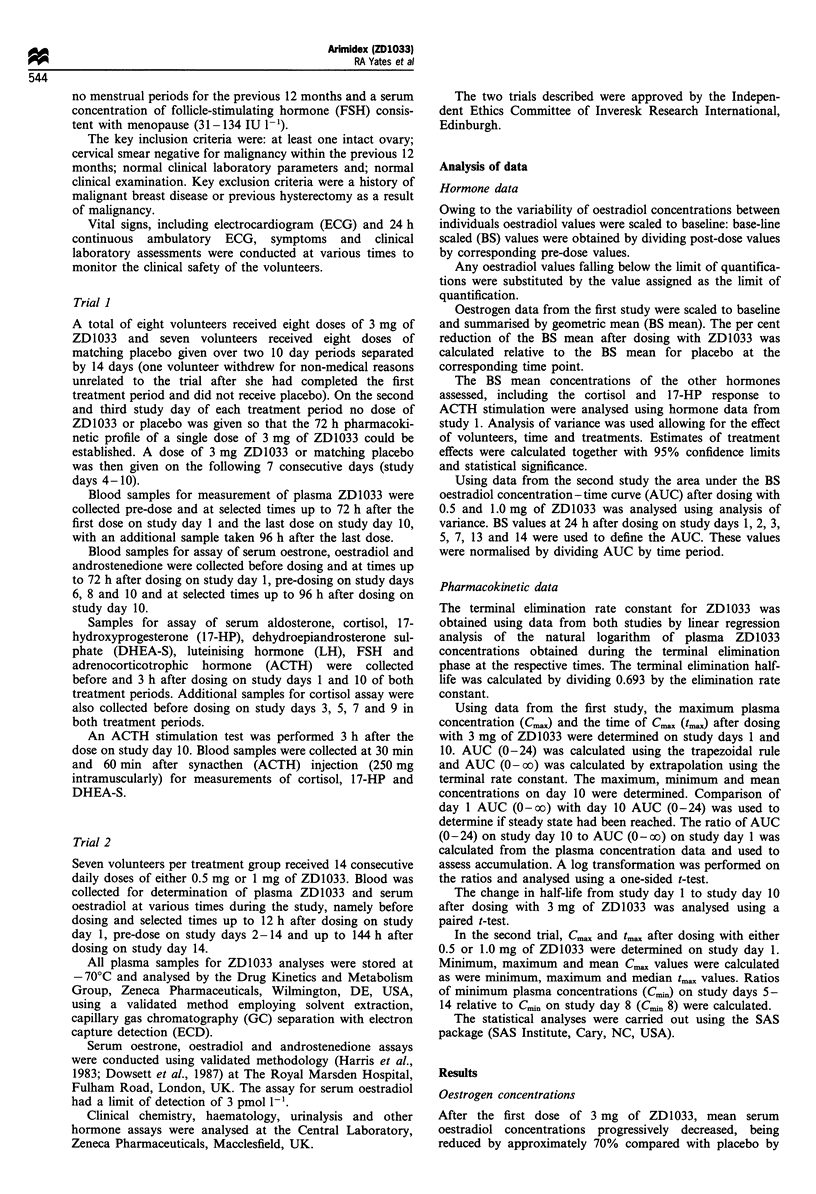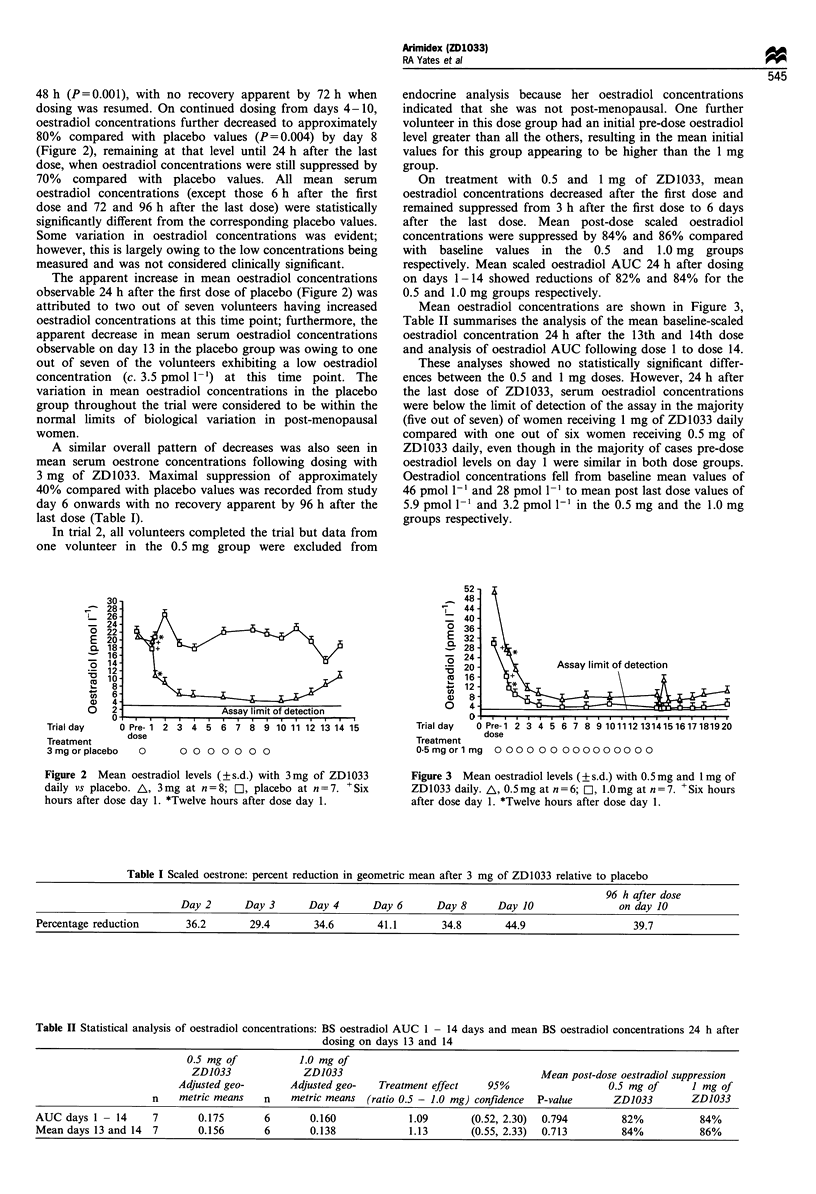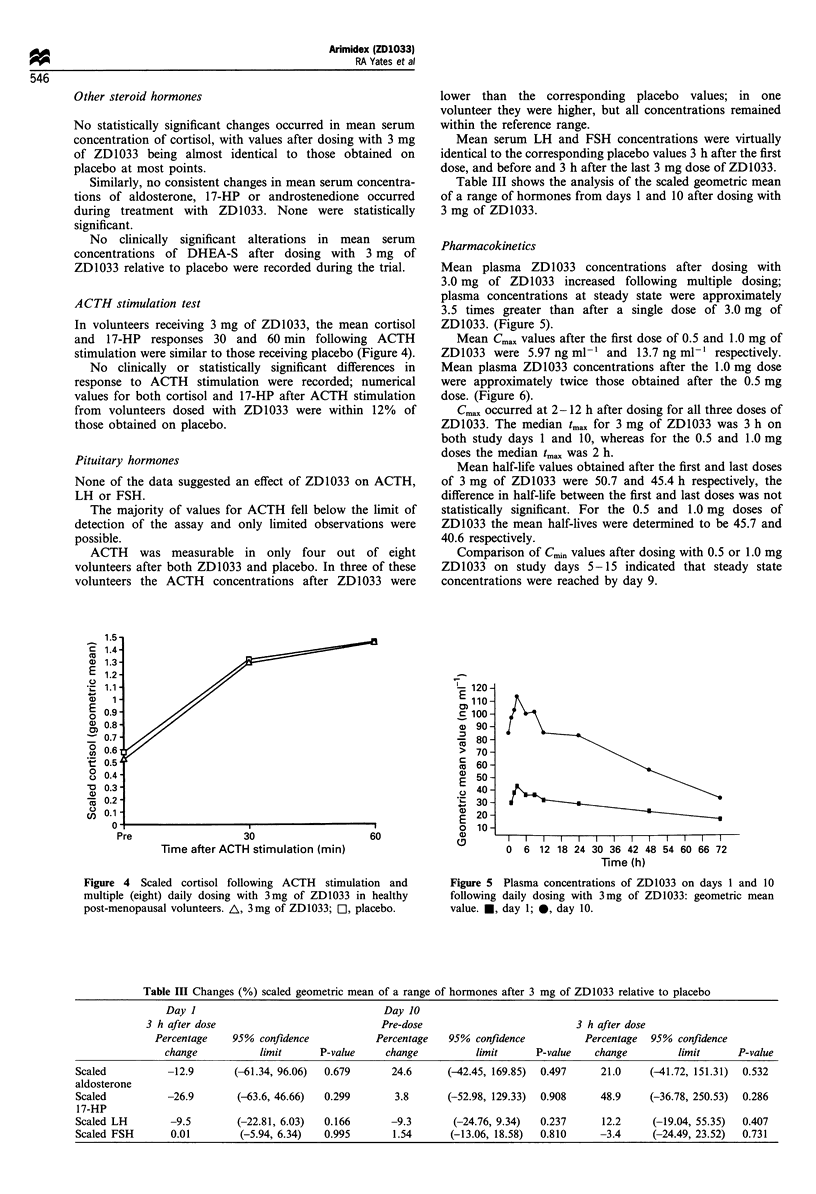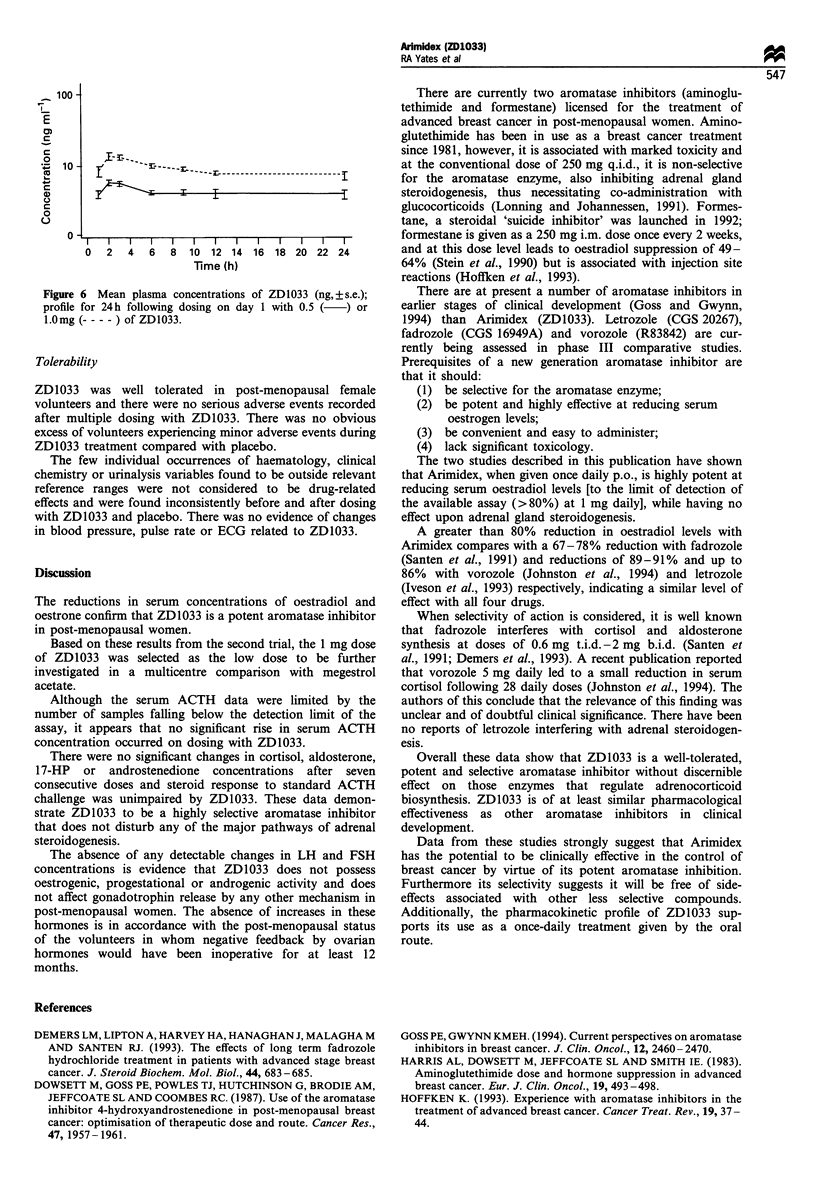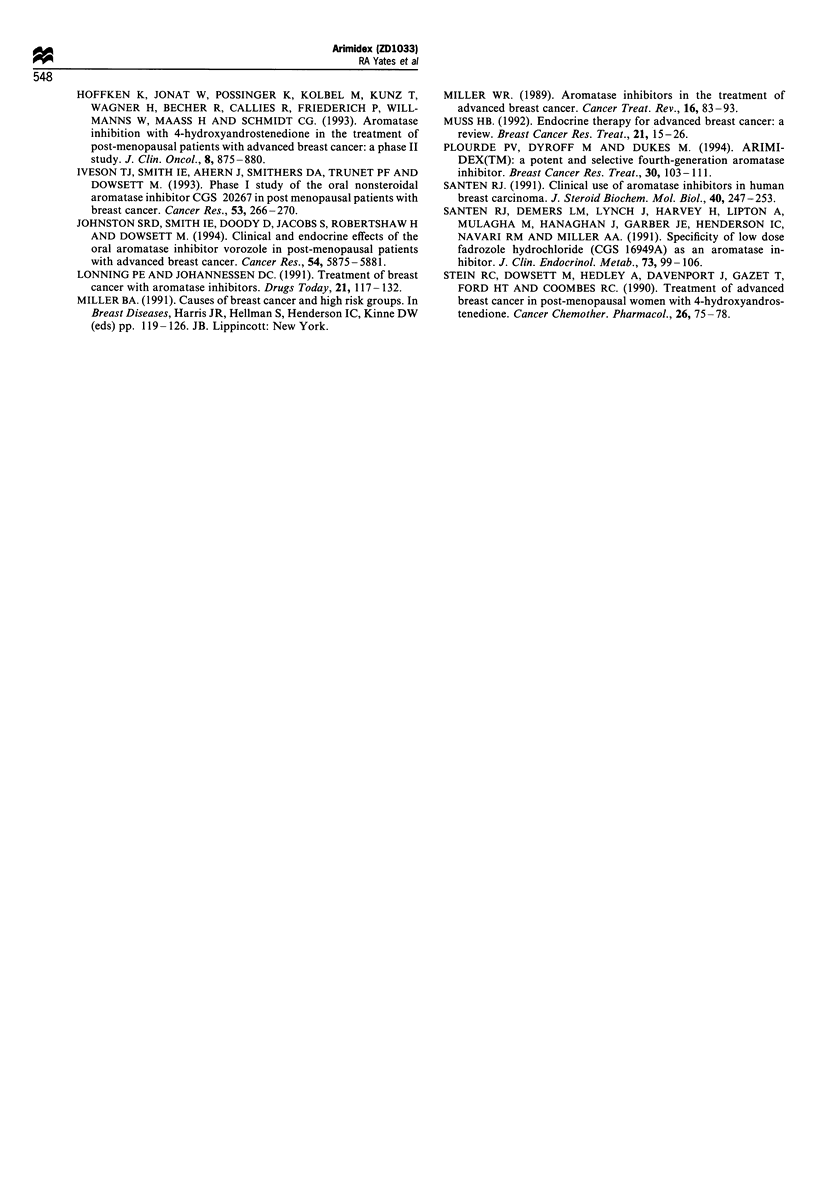# Arimidex (ZD1033): a selective, potent inhibitor of aromatase in postmenopausal female volunteers.

**DOI:** 10.1038/bjc.1996.94

**Published:** 1996-02

**Authors:** R. A. Yates, M. Dowsett, G. V. Fisher, A. Selen, P. J. Wyld

**Affiliations:** Zeneca Pharmaceuticals, Macclesfield, UK.

## Abstract

Two multiple-dose studies were conducted in healthy post-menopausal female volunteers to investigate the pharmacokinetics and effects on endocrinology of Arimidex (ZD1033). Volunteers in the first trial were dosed with 3 mg of ZD1033 daily over 10 days to assess the effects on endocrinology of ZD1033 and establish a pharmacokinetic profile. In the second trial volunteers received 14 daily doses of either 0.5 or 1.0 mg of ZD1033 to assess the pharmacokinetics of ZD1033 and the effects of low doses of ZD1033 on serum oestradiol concentrations. Following multiple dosing a significant reduction in the concentration of serum oestradiol of approximately 80% of baseline was obtained with all three doses; no recovery in oestradiol was apparent for up to 144 h after the last dose. There was no overall difference in the level of oestradiol suppression between the 0.5 or 1.0 mg doses of ZD1033. However, comparison of the number of volunteers with oestradiol concentrations below the limits of detection of the assay, 24 h after the last dose of ZD1033, suggested that 1.0 mg was the minimal dose required for maximal suppression of oestradiol. No significant effect was recorded on serum concentrations of gonadotrophins over the dosing period. Serum concentrations of a range of adrenal steroids were not affected by administration of ZD1033; furthermore, steroid response to standard adrenocorticotrophic hormone (ACTH) challenge was unimpaired by ZD1033. Together these data demonstrate the potency, tolerability and selectivity of ZD1033. The pharmacokinetic profile of ZD1033 supports its use as a once-daily treatment given orally.


British Journal of Cancer (1996) 73, 543-548
© 1996 Stockton Press All rights reserved 0007-0920/96 $12.00

### Arimidex (ZD1033): a selective, potent inhibitor of aromatase in post-menopausal female volunteers

RA Yates[1], M Dowsett[2], GV Fisher[1], A Selen[3] and PJ Wyld[4]

[1]*Zeneca Pharmaceuticals, Alderley Park, Macclesfield, Cheshire SK10 4TG, UK;* [2]*Royal Marsden Hospital, Fulham Road, London, SW3 6JJ, UK;* [3]*Zeneca Pharmaceuticals, 1800 Concord Pike, Wilmington, USA;* [4]*Inveresk Clinical Research Ltd, Riccarton, Edinburgh, UK.*

**Summary** Two multiple-dose studies were conducted in healthy post-menopausal female volunteers to investigate the pharmacokinetics and effects on endocrinology of Arimidex (ZD1033). Volunteers in the first trial were dosed with 3 mg of ZD1033 daily over 10 days to assess the effects on endocrinology of ZD1033 and establish the pharmacokinetic profile. In the second trial volunteers received 14 daily doses of either 0.5 or 1.0 mg of ZD1033 to assess the pharmacokinetics of ZD1033 and the effects of low doses of ZD1033 on serum oestradiol concentrations. Following multiple dosing a significant reduction in the concentration of serum oestradiol of approximately 80% of baseline was obtained with all three doses; no recovery in oestradiol was apparent for up to 144 h after the last dose. There was no overall difference in the level of oestradiol suppression between the 0.5 or 1.0 mg doses of ZD1033. However, comparison of the number of volunteers with oestradiol concentrations below the limits of detection of the assay, 24 h after the last dose of ZD1033, suggested that 1.0 mg was the minimal dose required for maximal suppression of oestradiol. No significant effect was recorded on serum concentrations of gonadotrophins over the dosing period. Serum concentrations of a range of adrenal steroids were not affected by administration of ZD1033; furthermore, steroid response to standard adrenocorticotrophic hormone (ACTH) challenge was unimpaired by ZD1033. Together these data demonstrate the potency, tolerability and selectivity of ZD1033. The pharmacokinetic profile of ZD1033 supports its use as a once-daily treatment given orally.

**Keywords:** Arimidex; aromatase inhibitor; oestrogen

Breast cancer is the most common type of cancer in women, accounting for 10% of all female cancers. It has been estimated that in the year 2000 the worldwide incidence will be between 1.1 and 1.4 million, whilst the mortality rate will be approximately 500 000–700 000 (Miller *et al.*, 1991).

Approximately one-third of human breast cancers are oestrogen dependent and will regress following oestrogen deprivation (Miller, 1989). In post-menopausal women the major mechanism for oestrogen production is the peripheral conversion (by aromatase) of the adrenal steroid androstenedione to oestrone and subsequently to oestradiol (Muss, 1992). Inhibition of aromatase therefore represents a promising method of treatment for post-menopausal women with breast cancer (Santen, 1991).

Aromatase inhibitors have been shown to be clinically effective in the treatment of advanced breast cancer (Hoffken, 1993). However the most widely used commercially available aromatase inhibitor, aminoglutethimide, is non-specific and inhibits several enzymes responsible for adrenocorticosteroid synthesis (Lonning and Johannessen, 1991). A second aromatase inhibitor, formestane, is inconvenient to use since it has to be given as a fortnightly intramuscular injection and has been reported to lead to injection site reactions (Hoffken *et al.*, 1993).

Several new non-steroidal aromatase inhibitors such as Arimidex (ZD1033) are currently undergoing clinical evaluation because they offer advantages over current aromatase inhibitors. ZD1033 is an achiral benzyltriazole derivative, 2,2[5-(1H-12,4-triazol-1-ylmethyl)-1,3-phenylene] *bis* (2-methyl-propriononitrile) (Figure 1).

Preclinical studies have shown that ZD1033 is a highly selective, potent aromatase inhibitor (Plourde *et al.*, 1994). ZD1033 is currently being evaluated in identical phase III efficacy and tolerability studies *vs* megestrol acetate in post-menopausal women with advanced breast cancer in the USA and Europe.

Correspondence: RA Yates
Received 29 March 1995; revised 28 August 1995; accepted 31 August 1995

This paper describes two studies that investigated the pharmacokinetic profile, selectivity, tolerability and efficacy in oestradiol suppression of multiple doses of ZD1033. The objective of the studies was to determine the minimum dose required for maximal suppression of circulating oestradiol, while retaining selectivity for the aromatase enzyme. The results of these studies were used as a basis for dose selection for the phase III efficacy studies.

### Methods

The two trials were randomised, double-blind, placebo-controlled multiple-dose studies in healthy post-menopausal female volunteers aged between 45 and 62 years (inclusive). Both trials had the same entry criteria and were of similar design.

In the first study (trial 1), a total of eight volunteers were given eight doses of 3 mg of ZD1033 over 10 days to assess tolerability, pharmacokinetics and effects on endocrinology. In the second study (trial 2) a total of 14 volunteers were given 0.5 and 1 mg doses of ZD1033 daily for 14 days to assess the tolerability, pharmacokinetics and effect on serum oestradiol concentrations only.

Post-menopausal status of the volunteers was defined as

**Figure 1** Structure of ZD1033.

no menstrual periods for the previous 12 months and a serum concentration of follicle-stimulating hormone (FSH) consistent with menopause (31–134 IU $l^{-1}$).

The key inclusion criteria were: at least one intact ovary; cervical smear negative for malignancy within the previous 12 months; normal clinical laboratory parameters and; normal clinical examination. Key exclusion criteria were a history of malignant breast disease or previous hysterectomy as a result of malignancy.

Vital signs, including electrocardiogram (ECG) and 24 h continuous ambulatory ECG, symptoms and clinical laboratory assessments were conducted at various times to monitor the clinical safety of the volunteers.

### *Trial 1*

A total of eight volunteers received eight doses of 3 mg of ZD1033 and seven volunteers received eight doses of matching placebo given over two 10 day periods separated by 14 days (one volunteer withdrew for non-medical reasons unrelated to the trial after she had completed the first treatment period and did not receive placebo). On the second and third study day of each treatment period no dose of ZD1033 or placebo was given so that the 72 h pharmacokinetic profile of a single dose of 3 mg of ZD1033 could be established. A dose of 3 mg ZD1033 or matching placebo was then given on the following 7 consecutive days (study days 4–10).

Blood samples for measurement of plasma ZD1033 were collected pre-dose and at selected times up to 72 h after the first dose on study day 1 and the last dose on study day 10, with an additional sample taken 96 h after the last dose.

Blood samples for assay of serum oestrone, oestradiol and androstenedione were collected before dosing and at times up to 72 h after dosing on study day 1, pre-dosing on study days 6, 8 and 10 and at selected times up to 96 h after dosing on study day 10.

Samples for assay of serum aldosterone, cortisol, 17-hydroxyprogesterone (17-HP), dehydroepiandrosterone sulphate (DHEA-S), luteinising hormone (LH), FSH and adrenocorticotrophic hormone (ACTH) were collected before and 3 h after dosing on study days 1 and 10 of both treatment periods. Additional samples for cortisol assay were also collected before dosing on study days 3, 5, 7 and 9 in both treatment periods.

An ACTH stimulation test was performed 3 h after the dose on study day 10. Blood samples were collected at 30 min and 60 min after synacthen (ACTH) injection (250 mg intramuscularly) for measurements of cortisol, 17-HP and DHEA-S.

### *Trial 2*

Seven volunteers per treatment group received 14 consecutive daily doses of either 0.5 mg or 1 mg of ZD1033. Blood was collected for determination of plasma ZD1033 and serum oestradiol at various times during the study, namely before dosing and selected times up to 12 h after dosing on study day 1, pre-dose on study days 2–14 and up to 144 h after dosing on study day 14.

All plasma samples for ZD1033 analyses were stored at −70°C and analysed by the Drug Kinetics and Metabolism Group, Zeneca Pharmaceuticals, Wilmington, DE, USA, using a validated method employing solvent extraction, capillary gas chromatography (GC) separation with electron capture detection (ECD).

Serum oestrone, oestradiol and androstenedione assays were conducted using validated methodology (Harris *et al.*, 1983; Dowsett *et al.*, 1987) at The Royal Marsden Hospital, Fulham Road, London, UK. The assay for serum oestradiol had a limit of detection of 3 pmol $l^{-1}$.

Clinical chemistry, haematology, urinalysis and other hormone assays were analysed at the Central Laboratory, Zeneca Pharmaceuticals, Macclesfield, UK.

The two trials described were approved by the Independent Ethics Committee of Inveresk Research International, Edinburgh.

### Analysis of data

### *Hormone data*

Owing to the variability of oestradiol concentrations between individuals oestradiol values were scaled to baseline: base-line scaled (BS) values were obtained by dividing post-dose values by corresponding pre-dose values.

Any oestradiol values falling below the limit of quantifications were substituted by the value assigned as the limit of quantification.

Oestrogen data from the first study were scaled to baseline and summarised by geometric mean (BS mean). The per cent reduction of the BS mean after dosing with ZD1033 was calculated relative to the BS mean for placebo at the corresponding time point.

The BS mean concentrations of the other hormones assessed, including the cortisol and 17-HP response to ACTH stimulation were analysed using hormone data from study 1. Analysis of variance was used allowing for the effect of volunteers, time and treatments. Estimates of treatment effects were calculated together with 95% confidence limits and statistical significance.

Using data from the second study the area under the BS oestradiol concentration–time curve (AUC) after dosing with 0.5 and 1.0 mg of ZD1033 was analysed using analysis of variance. BS values at 24 h after dosing on study days 1, 2, 3, 5, 7, 13 and 14 were used to define the AUC. These values were normalised by dividing AUC by time period.

### *Pharmacokinetic data*

The terminal elimination rate constant for ZD1033 was obtained using data from both studies by linear regression analysis of the natural logarithm of plasma ZD1033 concentrations obtained during the terminal elimination phase at the respective times. The terminal elimination half-life was calculated by dividing 0.693 by the elimination rate constant.

Using data from the first study, the maximum plasma concentration ($C_{max}$) and the time of $C_{max}$ ($t_{max}$) after dosing with 3 mg of ZD1033 were determined on study days 1 and 10. AUC (0–24) was calculated using the trapezoidal rule and AUC (0–∞) was calculated by extrapolation using the terminal rate constant. The maximum, minimum and mean concentrations on day 10 were determined. Comparison of day 1 AUC (0–∞) with day 10 AUC (0–24) was used to determine if steady state had been reached. The ratio of AUC (0–24) on study day 10 to AUC (0–∞) on study day 1 was calculated from the plasma concentration data and used to assess accumulation. A log transformation was performed on the ratios and analysed using a one-sided $t$-test.

The change in half-life from study day 1 to study day 10 after dosing with 3 mg of ZD1033 was analysed using a paired $t$-test.

In the second trial, $C_{max}$ and $t_{max}$ after dosing with either 0.5 or 1.0 mg of ZD1033 were determined on study day 1. Minimum, maximum and mean $C_{max}$ values were calculated as were minimum, maximum and median $t_{max}$ values. Ratios of minimum plasma concentrations ($C_{min}$) on study days 5–14 relative to $C_{min}$ on study day 8 ($C_{min}$ 8) were calculated.

The statistical analyses were carried out using the SAS package (SAS Institute, Cary, NC, USA).

### Results

### *Oestrogen concentrations*

After the first dose of 3 mg of ZD1033, mean serum oestradiol concentrations progressively decreased, being reduced by approximately 70% compared with placebo by

48 h ($P=0.001$), with no recovery apparent by 72 h when dosing was resumed. On continued dosing from days 4–10, oestradiol concentrations further decreased to approximately 80% compared with placebo values ($P=0.004$) by day 8 (Figure 2), remaining at that level until 24 h after the last dose, when oestradiol concentrations were still suppressed by 70% compared with placebo values. All mean serum oestradiol concentrations (except those 6 h after the first dose and 72 and 96 h after the last dose) were statistically significantly different from the corresponding placebo values. Some variation in oestradiol concentrations was evident; however, this is largely owing to the low concentrations being measured and was not considered clinically significant.

The apparent increase in mean oestradiol concentrations observable 24 h after the first dose of placebo (Figure 2) was attributed to two out of seven volunteers having increased oestradiol concentrations at this time point; furthermore, the apparent decrease in mean serum oestradiol concentrations observable on day 13 in the placebo group was owing to one out of seven of the volunteers exhibiting a low oestradiol concentration (*c.* 3.5 pmol $l^{-1}$) at this time point. The variation in mean oestradiol concentrations in the placebo group throughout the trial were considered to be within the normal limits of biological variation in post-menopausal women.

A similar overall pattern of decreases was also seen in mean serum oestrone concentrations following dosing with 3 mg of ZD1033. Maximal suppression of approximately 40% compared with placebo values was recorded from study day 6 onwards with no recovery apparent by 96 h after the last dose (Table I).

In trial 2, all volunteers completed the trial but data from one volunteer in the 0.5 mg group were excluded from endocrine analysis because her oestradiol concentrations indicated that she was not post-menopausal. One further volunteer in this dose group had an initial pre-dose oestradiol level greater than all the others, resulting in the mean initial values for this group appearing to be higher than the 1 mg group.

On treatment with 0.5 and 1 mg of ZD1033, mean oestradiol concentrations decreased after the first dose and remained suppressed from 3 h after the first dose to 6 days after the last dose. Mean post-dose scaled oestradiol concentrations were suppressed by 84% and 86% compared with baseline values in the 0.5 and 1.0 mg groups respectively. Mean scaled oestradiol AUC 24 h after dosing on days 1–14 showed reductions of 82% and 84% for the 0.5 and 1.0 mg groups respectively.

Mean oestradiol concentrations are shown in Figure 3, Table II summarises the analysis of the mean baseline-scaled oestradiol concentration 24 h after the 13th and 14th dose and analysis of oestradiol AUC following dose 1 to dose 14.

These analyses showed no statistically significant differences between the 0.5 and 1 mg doses. However, 24 h after the last dose of ZD1033, serum oestradiol concentrations were below the limit of detection of the assay in the majority (five out of seven) of women receiving 1 mg of ZD1033 daily compared with one out of six women receiving 0.5 mg of ZD1033 daily, even though in the majority of cases pre-dose oestradiol levels on day 1 were similar in both dose groups. Oestradiol concentrations fell from baseline mean values of 46 pmol $l^{-1}$ and 28 pmol $l^{-1}$ to mean post last dose values of 5.9 pmol $l^{-1}$ and 3.2 pmol $l^{-1}$ in the 0.5 mg and the 1.0 mg groups respectively.

**Figure 2** Mean oestradiol levels (±s.d.) with 3 mg of ZD1033 daily *vs* placebo. △, 3 mg at $n=8$; □, placebo at $n=7$. +Six hours after dose day 1. *Twelve hours after dose day 1.

**Figure 3** Mean oestradiol levels (±s.d.) with 0.5 mg and 1 mg of ZD1033 daily. △, 0.5 mg at $n=6$; □, 1.0 mg at $n=7$. +Six hours after dose day 1. *Twelve hours after dose day 1.

**Table I** Scaled oestrone: percent reduction in geometric mean after 3 mg of ZD1033 relative to placebo

| | *Day 2* | *Day 3* | *Day 4* | *Day 6* | *Day 8* | *Day 10* | *96 h after dose on day 10* |
|---|---|---|---|---|---|---|---|
| Percentage reduction | 36.2 | 29.4 | 34.6 | 41.1 | 34.8 | 44.9 | 39.7 |

**Table II** Statistical analysis of oestradiol concentrations: BS oestradiol AUC 1 – 14 days and mean BS oestradiol concentrations 24 h after dosing on days 13 and 14

| | n | *0.5 mg of ZD1033 Adjusted geometric means* | n | *1.0 mg of ZD1033 Adjusted geometric means* | *Treatment effect (ratio 0.5 – 1.0 mg)* | *95% confidence* | P-*value* | *Mean post-dose oestradiol suppression 0.5 mg of ZD1033* | *Mean post-dose oestradiol suppression 1 mg of ZD1033* |
|---|---|---|---|---|---|---|---|---|---|
| AUC days 1 – 14 | 7 | 0.175 | 6 | 0.160 | 1.09 | (0.52, 2.30) | 0.794 | 82% | 84% |
| Mean days 13 and 14 | 7 | 0.156 | 6 | 0.138 | 1.13 | (0.55, 2.33) | 0.713 | 84% | 86% |

### *Other steroid hormones*

No statistically significant changes occurred in mean serum concentration of cortisol, with values after dosing with 3 mg of ZD1033 being almost identical to those obtained on placebo at most points.

Similarly, no consistent changes in mean serum concentrations of aldosterone, 17-HP or androstenedione occurred during treatment with ZD1033. None were statistically significant.

No clinically significant alterations in mean serum concentrations of DHEA-S after dosing with 3 mg of ZD1033 relative to placebo were recorded during the trial.

### *ACTH stimulation test*

In volunteers receiving 3 mg of ZD1033, the mean cortisol and 17-HP responses 30 and 60 min following ACTH stimulation were similar to those receiving placebo (Figure 4).

No clinically or statistically significant differences in response to ACTH stimulation were recorded; numerical values for both cortisol and 17-HP after ACTH stimulation from volunteers dosed with ZD1033 were within 12% of those obtained on placebo.

### *Pituitary hormones*

None of the data suggested an effect of ZD1033 on ACTH, LH or FSH.

The majority of values for ACTH fell below the limit of detection of the assay and only limited observations were possible.

ACTH was measurable in only four out of eight volunteers after both ZD1033 and placebo. In three of these volunteers the ACTH concentrations after ZD1033 were lower than the corresponding placebo values; in one volunteer they were higher, but all concentrations remained within the reference range.

Mean serum LH and FSH concentrations were virtually identical to the corresponding placebo values 3 h after the first dose, and before and 3 h after the last 3 mg dose of ZD1033.

Table III shows the analysis of the scaled geometric mean of a range of hormones from days 1 and 10 after dosing with 3 mg of ZD1033.

### *Pharmacokinetics*

Mean plasma ZD1033 concentrations after dosing with 3.0 mg of ZD1033 increased following multiple dosing; plasma concentrations at steady state were approximately 3.5 times greater than after a single dose of 3.0 mg of ZD1033. (Figure 5).

Mean $C_{max}$ values after the first dose of 0.5 and 1.0 mg of ZD1033 were 5.97 ng ml$^{-1}$ and 13.7 ng ml$^{-1}$ respectively. Mean plasma ZD1033 concentrations after the 1.0 mg dose were approximately twice those obtained after the 0.5 mg dose. (Figure 6).

$C_{max}$ occurred at 2–12 h after dosing for all three doses of ZD1033. The median $t_{max}$ for 3 mg of ZD1033 was 3 h on both study days 1 and 10, whereas for the 0.5 and 1.0 mg doses the median $t_{max}$ was 2 h.

Mean half-life values obtained after the first and last doses of 3 mg of ZD1033 were 50.7 and 45.4 h respectively, the difference in half-life between the first and last doses was not statistically significant. For the 0.5 and 1.0 mg doses of ZD1033 the mean half-lives were determined to be 45.7 and 40.6 respectively.

Comparison of $C_{min}$ values after dosing with 0.5 or 1.0 mg ZD1033 on study days 5–15 indicated that steady state concentrations were reached by day 9.

**Figure 4** Scaled cortisol following ACTH stimulation and multiple (eight) daily dosing with 3 mg of ZD1033 in healthy post-menopausal volunteers. △, 3 mg of ZD1033; □, placebo.

**Figure 5** Plasma concentrations of ZD1033 on days 1 and 10 following daily dosing with 3 mg of ZD1033: geometric mean value. ■, day 1; ●, day 10.

**Table III** Changes (%) scaled geometric mean of a range of hormones after 3 mg of ZD1033 relative to placebo

| | *Day 1 3 h after dose Percentage change* | *95% confidence limit* | *P-value* | *Day 10 Pre-dose Percentage change* | *95% confidence limit* | *P-value* | *3 h after dose Percentage change* | *95% confidence limit* | *P-value* |
|---|---|---|---|---|---|---|---|---|---|
| Scaled aldosterone | −12.9 | (−61.34, 96.06) | 0.679 | 24.6 | (−42.45, 169.85) | 0.497 | 21.0 | (−41.72, 151.31) | 0.532 |
| Scaled 17-HP | −26.9 | (−63.6, 46.66) | 0.299 | 3.8 | (−52.98, 129.33) | 0.908 | 48.9 | (−36.78, 250.53) | 0.286 |
| Scaled LH | −9.5 | (−22.81, 6.03) | 0.166 | −9.3 | (−24.76, 9.34) | 0.237 | 12.2 | (−19.04, 55.35) | 0.407 |
| Scaled FSH | 0.01 | (−5.94, 6.34) | 0.995 | 1.54 | (−13.06, 18.58) | 0.810 | −3.4 | (−24.49, 23.52) | 0.731 |

**Figure 6** Mean plasma concentrations of ZD1033 (ng, ±s.e.); profile for 24 h following dosing on day 1 with 0.5 (——) or 1.0 mg (- - - - ) of ZD1033.

*Tolerability*

ZD1033 was well tolerated in post-menopausal female volunteers and there were no serious adverse events recorded after multiple dosing with ZD1033. There was no obvious excess of volunteers experiencing minor adverse events during ZD1033 treatment compared with placebo.

The few individual occurrences of haematology, clinical chemistry or urinalysis variables found to be outside relevant reference ranges were not considered to be drug-related effects and were found inconsistently before and after dosing with ZD1033 and placebo. There was no evidence of changes in blood pressure, pulse rate or ECG related to ZD1033.

### Discussion

The reductions in serum concentrations of oestradiol and oestrone confirm that ZD1033 is a potent aromatase inhibitor in post-menopausal women.

Based on these results from the second trial, the 1 mg dose of ZD1033 was selected as the low dose to be further investigated in a multicentre comparison with megestrol acetate.

Although the serum ACTH data were limited by the number of samples falling below the detection limit of the assay, it appears that no significant rise in serum ACTH concentration occurred on dosing with ZD1033.

There were no significant changes in cortisol, aldosterone, 17-HP or androstenedione concentrations after seven consecutive doses and steroid response to standard ACTH challenge was unimpaired by ZD1033. These data demonstrate ZD1033 to be a highly selective aromatase inhibitor that does not disturb any of the major pathways of adrenal steroidogenesis.

The absence of any detectable changes in LH and FSH concentrations is evidence that ZD1033 does not possess oestrogenic, progestational or androgenic activity and does not affect gonadotrophin release by any other mechanism in post-menopausal women. The absence of increases in these hormones is in accordance with the post-menopausal status of the volunteers in whom negative feedback by ovarian hormones would have been inoperative for at least 12 months.

There are currently two aromatase inhibitors (aminoglutethimide and formestane) licensed for the treatment of advanced breast cancer in post-menopausal women. Aminoglutethimide has been in use as a breast cancer treatment since 1981, however, it is associated with marked toxicity and at the conventional dose of 250 mg q.i.d., it is non-selective for the aromatase enzyme, also inhibiting adrenal gland steroidogenesis, thus necessitating co-administration with glucocorticoids (Lonning and Johannessen, 1991). Formestane, a steroidal 'suicide inhibitor' was launched in 1992; formestane is given as a 250 mg i.m. dose once every 2 weeks, and at this dose level leads to oestradiol suppression of 49–64% (Stein *et al.*, 1990) but is associated with injection site reactions (Hoffken *et al.*, 1993).

There are at present a number of aromatase inhibitors in earlier stages of clinical development (Goss and Gwynn, 1994) than Arimidex (ZD1033). Letrozole (CGS 20267), fadrozole (CGS 16949A) and vorozole (R83842) are currently being assessed in phase III comparative studies. Prerequisites of a new generation aromatase inhibitor are that it should:

(1) be selective for the aromatase enzyme;
(2) be potent and highly effective at reducing serum oestrogen levels;
(3) be convenient and easy to administer;
(4) lack significant toxicology.

The two studies described in this publication have shown that Arimidex, when given once daily p.o., is highly potent at reducing serum oestradiol levels [to the limit of detection of the available assay (>80%) at 1 mg daily], while having no effect upon adrenal gland steroidogenesis.

A greater than 80% reduction in oestradiol levels with Arimidex compares with a 67–78% reduction with fadrozole (Santen *et al.*, 1991) and reductions of 89–91% and up to 86% with vorozole (Johnston *et al.*, 1994) and letrozole (Iveson *et al.*, 1993) respectively, indicating a similar level of effect with all four drugs.

When selectivity of action is considered, it is well known that fadrozole interferes with cortisol and aldosterone synthesis at doses of 0.6 mg t.i.d.–2 mg b.i.d. (Santen *et al.*, 1991; Demers *et al.*, 1993). A recent publication reported that vorozole 5 mg daily led to a small reduction in serum cortisol following 28 daily doses (Johnston *et al.*, 1994). The authors of this conclude that the relevance of this finding was unclear and of doubtful clinical significance. There have been no reports of letrozole interfering with adrenal steroidogenesis.

Overall these data show that ZD1033 is a well-tolerated, potent and selective aromatase inhibitor without discernible effect on those enzymes that regulate adrenocorticoid biosynthesis. ZD1033 is of at least similar pharmacological effectiveness as other aromatase inhibitors in clinical development.

Data from these studies strongly suggest that Arimidex has the potential to be clinically effective in the control of breast cancer by virtue of its potent aromatase inhibition. Furthermore its selectivity suggests it will be free of side-effects associated with other less selective compounds. Additionally, the pharmacokinetic profile of ZD1033 supports its use as a once-daily treatment given by the oral route.

### References

DEMERS LM, LIPTON A, HARVEY HA, HANAGHAN J, MALAGHA M AND SANTEN RJ. (1993). The effects of long term fadrozole hydrochloride treatment in patients with advanced stage breast cancer. *J. Steroid Biochem. Mol. Biol.*, **44**, 683–685.

DOWSETT M, GOSS PE, POWLES TJ, HUTCHINSON G, BRODIE AM, JEFFCOATE SL AND COOMBES RC. (1987). Use of the aromatase inhibitor 4-hydroxyandrostenedione in post-menopausal breast cancer: optimisation of therapeutic dose and route. *Cancer Res.*, **47**, 1957–1961.

GOSS PE, GWYNN KMEH. (1994). Current perspectives on aromatase inhibitors in breast cancer. *J. Clin. Oncol.*, **12**, 2460–2470.

HARRIS AL, DOWSETT M, JEFFCOATE SL AND SMITH IE. (1983). Aminoglutethimide dose and hormone suppression in advanced breast cancer. *Eur. J. Clin. Oncol.*, **19**, 493–498.

HOFFKEN K. (1993). Experience with aromatase inhibitors in the treatment of advanced breast cancer. *Cancer Treat. Rev.*, **19**, 37–44.

HOFFKEN K, JONAT W, POSSINGER K, KOLBEL M, KUNZ T, WAGNER H, BECHER R, CALLIES R, FRIEDERICH P, WILLMANNS W, MAASS H AND SCHMIDT CG. (1993). Aromatase inhibition with 4-hydroxyandrostenedione in the treatment of post-menopausal patients with advanced breast cancer: a phase II study. *J. Clin. Oncol.*, **8**, 875–880.

IVESON TJ, SMITH IE, AHERN J, SMITHERS DA, TRUNET PF AND DOWSETT M. (1993). Phase I study of the oral nonsteroidal aromatase inhibitor CGS 20267 in post menopausal patients with breast cancer. *Cancer Res.*, **53**, 266–270.

JOHNSTON SRD, SMITH IE, DOODY D, JACOBS S, ROBERTSHAW H AND DOWSETT M. (1994). Clinical and endocrine effects of the oral aromatase inhibitor vorozole in post-menopausal patients with advanced breast cancer. *Cancer Res.*, **54**, 5875–5881.

LONNING PE AND JOHANNESSEN DC. (1991). Treatment of breast cancer with aromatase inhibitors. *Drugs Today*, **21**, 117–132.

MILLER BA. (1991). Causes of breast cancer and high risk groups. In *Breast Diseases*, Harris JR, Hellman S, Henderson IC, Kinne DW (eds) pp. 119–126. JB. Lippincott: New York.

MILLER WR. (1989). Aromatase inhibitors in the treatment of advanced breast cancer. *Cancer Treat. Rev.*, **16**, 83–93.

MUSS HB. (1992). Endocrine therapy for advanced breast cancer: a review. *Breast Cancer Res. Treat.*, **21**, 15–26.

PLOURDE PV, DYROFF M AND DUKES M. (1994). ARIMIDEX(TM): a potent and selective fourth-generation aromatase inhibitor. *Breast Cancer Res. Treat.*, **30**, 103–111.

SANTEN RJ. (1991). Clinical use of aromatase inhibitors in human breast carcinoma. *J. Steroid Biochem. Mol. Biol.*, **40**, 247–253.

SANTEN RJ, DEMERS LM, LYNCH J, HARVEY H, LIPTON A, MULAGHA M, HANAGHAN J, GARBER JE, HENDERSON IC, NAVARI RM AND MILLER AA. (1991). Specificity of low dose fadrozole hydrochloride (CGS 16949A) as an aromatase inhibitor. *J. Clin. Endocrinol. Metab.*, **73**, 99–106.

STEIN RC, DOWSETT M, HEDLEY A, DAVENPORT J, GAZET T, FORD HT AND COOMBES RC. (1990). Treatment of advanced breast cancer in post-menopausal women with 4-hydroxyandrostenedione. *Cancer Chemother. Pharmacol.*, **26**, 75–78.